# A Novel Maternally Inherited *GNAS* Variant in a Family With Hyperphagia and Obesity: 3 Cases

**DOI:** 10.1210/jcemcr/luae125

**Published:** 2024-08-05

**Authors:** Anand Ramakrishnan, Dillon Popat, Preetha Purushothaman, Li F Chan, Evelien F Gevers

**Affiliations:** Department of Paediatric Endocrinology, Barts Health NHS Trust—Royal London Children's Hospital, Whitechapel Road, London E1 1FR, UK; Centre for Endocrinology, William Harvey Research Institute, Faculty of Medicine and Dentistry, Queen Mary University of London, Charterhouse Square, EC1M 6BQ, London, UK; Department of Paediatric Endocrinology, Barts Health NHS Trust—Royal London Children's Hospital, Whitechapel Road, London E1 1FR, UK; Department of Paediatric Endocrinology, Barts Health NHS Trust—Royal London Children's Hospital, Whitechapel Road, London E1 1FR, UK; Centre for Endocrinology, William Harvey Research Institute, Faculty of Medicine and Dentistry, Queen Mary University of London, Charterhouse Square, EC1M 6BQ, London, UK; Department of Paediatric Endocrinology, Barts Health NHS Trust—Royal London Children's Hospital, Whitechapel Road, London E1 1FR, UK; Centre for Endocrinology, William Harvey Research Institute, Faculty of Medicine and Dentistry, Queen Mary University of London, Charterhouse Square, EC1M 6BQ, London, UK

**Keywords:** obesity, GNAS, iPPSD2, pseudohypoparathyroidism, PTH resistance, genetics of obesity

## Abstract

*GNAS* variants were recently described in 1% of patients not known to have pseudohypoparathyroidism/inactivating PTH/PTHrP signalling disorder 2 in the UK Genetics of Obesity Study. We describe a new missense *GNAS* variant, c.791A > C, p.(Asp264Thr), in a family with obesity, hyperphagia and mild PTH resistance. A 6-year-old female (body mass index +4.3 SD score [SDS], height +1.9 SDS) presented with hyperphagia and obesity from age 3 years. She had subtle brachydactyly, macrocephaly, and mildly delayed development. The 12-year-old brother (height +2.1 SDS, body mass index +2.9 SDS) had hyperphagia, obesity, mildly delayed development, and autism. He had subtle brachydactyly, as did the affected mother. We assessed the functional effect of the mutant, measuring cAMP production in cells transfected with wild type and mutant GNAS after ligand stimulation. Cells with the mutant GNAS showed impaired cAMP generation through melanocortin receptor 4, GH releasing hormone receptor, and PTH receptor. These cases demonstrate the clinical heterogeneity of monogenic disease, suggesting a need to test for PHP1A in children with obesity even without classical signs of PHP1A.

## Introduction

Pseudohypoparathyroidism type 1A (PHP1A/iPPSD2) is characterized by brachydactyly, short stature, round face, stocky appearance, ectopic calcifications (Albright hereditary osteodystrophy [AHO]), developmental delay, and resistance to PTH or other hormones binding G-protein coupled receptors (GPCR) (1). PHP1A/IPPSD2 occurs because of mutations in the maternal allele of the *GNAS* gene, resulting in impaired signalling via GPCRs.

Recently, *GNAS* variants were described in 22 of 2548 patients with severe early-onset obesity (Genetics of Obesity Study [GOOS]), not previously noted to have PHP (2). These mutations impaired melanocortin 4 receptor (MC4R) signalling, a major regulator of appetite. We report another *GNAS* variant in 2 patients referred for obesity and their mother. Functional analysis shows impaired signalling in vitro after ligand stimulation of MC4R, GHRHR, and PTHR. The patients have hyperphagia and obesity but only mild brachydactyly and PTH resistance.

## Case Presentation

### Case 1 (Proband)

**Figure 1.**
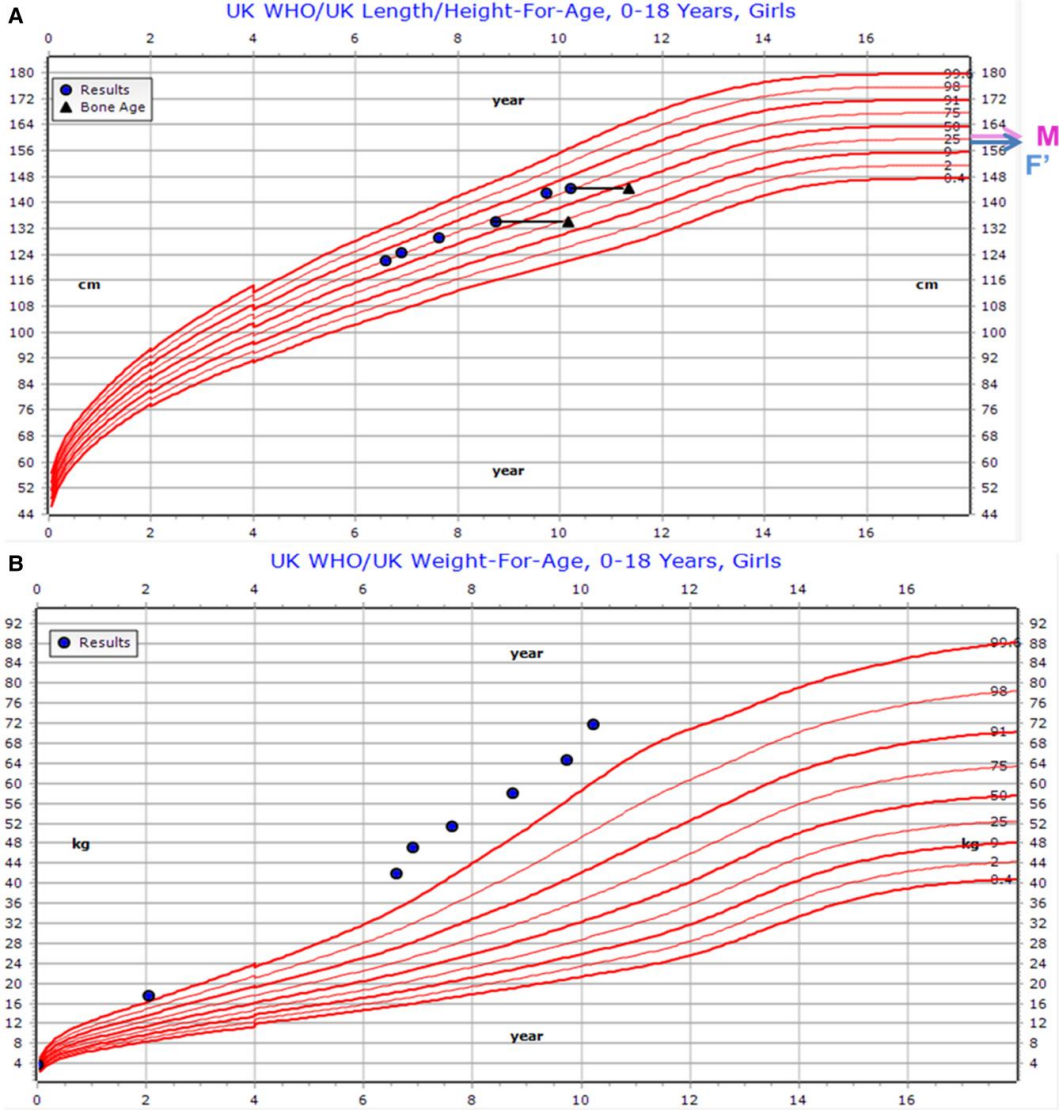
Growth charts depicting height (A) and weight (B) for case 1. Triangles in the height chart depict bone age. X axis represents age in years. Y axis represents height (cm) or weight (kg). M indicates height of mother and F′ indicates height of father, corrected for sex.

A 6-year-old female, born to nonconsanguineous parents of Northern African origin, presented with hyperphagia and weight gain from 3 years of age. Birth weight was 3.79 kg (+1.1 SD score [SDS]). She walked at 15 months of age, talked at 3 years, and needs additional help at school. At presentation (aged 6.4 years), her weight was 47.1 kg (+3.7 SDS), body mass index (BMI) 30.39 kg/m^2^ (+4.0 SDS), height 124.9 cm (+1.9 SDS) and head circumference (HC) 56.0 cm (+3.0 SDS) (Fig. 1). Her mother was 160.2 cm (−0.6 SDS) and father was 170.5 cm (−1.1 SDS). She had subtle brachydactyly with slightly short fourth and fifth metacarpals, short toes, broad great toe, macrocephaly, and a café au lait patch on the chest. Her BMI reduced when she had dietary support at school with increased physical activity (aged 8.75-9.75 years) (32.2 kg/m^2^, +3.7 SDS to 31.7 kg/m^2^, +3.5 SDS) but gained 7 kg rapidly once this was discontinued (Fig. 1).

#### Investigations

**Figure 2.**
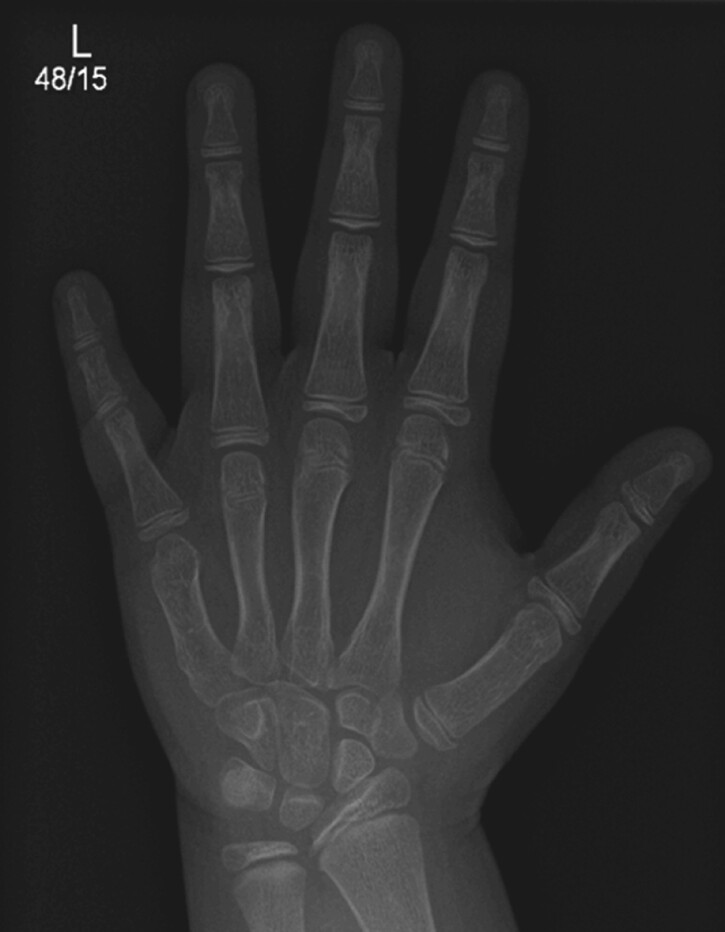
X-ray of the hand and wrist of case 1 showing mild brachydactyly with short 5th metacarpal.

Biochemical investigations are shown in Table 1. Initial PTH was 9.5 pmol/L (0.7-5.6) (89.6 pg/mL [6.6-52.8]) and subsequently increased to 16.6 pmol/L (156.5 pg/mL) with normal calcium, phosphorus, and vitamin D. There was no evidence of TSH or GH resistance. She developed hypercholesterolemia and diffuse fatty liver. Bone age at 8.7 years and 10.2 years was advanced by 1.5 and 1.1 years (Tanner and Whitehouse 3). Hand X ray showed mild brachydactyly with a short right fifth metacarpal (Fig. 2).

**Table 1.** Comparison of clinical and biochemical characteristics of the 3 patients

Feature	Case 1 (proband)	Case 2 (proband's brother)	Case 3 (proband's mother)
Obesity	+++	+++	+++
Hyperphagia	++	++	—
Brachydactyly	+	+	+
Macrocephaly	+	—	—
Ectopic ossification	—	—	—
Learning difficulty	+	+	—
Cafe au lait macule	+	—	—
PTH resistance	+	+	+
Hypocalcaemia	—	—	+
TSH resistance	—	—	—
TSH (<6.0 mU/L)	4.2 mU/L (6.7 y)	4.2 mU/L (11.1 y)	3.5 mU/L
	6.6 mU/L (8.6 y)	4.0 mU/L (12.9 y)	
	6.0 mU/L (9.7 y)	4.5 mU/L (13.3 y)	
	3.8 mU/L (10.2 y)		
PTH (1.6-6.9 pmol/L) (6.6-52.8 pg/mL)	9.6 pmol/L (8.6 y) (89.6 pg/mL)	18.4 pmol/L (12.9 y) (173.5 pg/mL)	10.7 pmol/L (100.9 pg/mL)
	16.6 pmol/L (9.7 y) (156.5 pg/mL)	15.3 pmol/L (13.3 y) (144.3 pg/mL)	
	16.6 pmol/L (10.2 y) (156.5 pg/mL)		
Calcium (2.2-2.6 mmol/L) (8.8-10.4 mg/dL)	2.38 mmol/L (9.5 mg/dL)	2.24 mmol/L (9.0 mg/dL)	2.18 mmol/L (8.7 mg/dL)
Phosphorus Female, 6 y of age (Case 1) (0.9-1.8 mmol/L) (2.8-5.6 mg/dL) Male, 12 y of age (Case 2) (0.9-1.8 mmol/L) (2.8-5.6 mg/dL) Female, 37 y of age (Case 3) (0.8-1.5 mmol/L) (2.5-4.6 mg/dL)	1.8 mmol/L (5.6 mg/dL)	1.5 mmol/L (4.6 mg/dL)	1.2 mmol/L (3.7 mg/dL)
Vitamin D (>25 nmol/L) (>10 ng/mL)	55 nmol/L (22 ng/mL)	50 nmol/L (20 ng/mL)	49 nmol/L (19.6 ng/mL)
IGF-1 Female, 6 y of age (Case 1) (7.2-62.3 nmol/L) (55-477 mcg/L) Male, 12 y of age (Case 2) (8.7-65 nmol/L) (67-498 mcg/L) Female, 37 y of age (Case 3) (10.5-36.3 nmol/L) (81-278 mcg/L)	16.6 nmol/L (127 mcg/L)	47.2 nmol/L (361 mcg/L)	17.7 nmol/L 136 mcg/L
LH (Unit/L) Prepubertal <1.0 Follicular 2.4-12.6 Ovulation 14-95.6 Luteal 1.0-11.4	<1.0 Unit/L (10.0 y)	11.3 Unit/L (13.3 y)	6.0 Unit/L
FSH (Unit/L) Prepubertal <1.0 Follicular 3.5-12.5 Ovulation 4.7-21.5 Luteal 1.7-7.7	1.6 Unit/L (10.0 y)	6.5 Unit/L (13.3 y)	5.0 Unit/L
Estradiol Prepubertal (<19 pmol/L) (<5.2 pg/ml) Follicular (45-854 pmol/L) (12.3-232.6 pg/mL) Ovulation (151-1461 pmol/L) (41.1-397.9 pg/mL) Luteal (82-1251 pmol/L) (22.3-340.7 pg/mL)	<19 pmol/L (10.0 y) (<5.2 pg/mL)	6.9 nmol/L (13.3 y) (199 ng/dL)	462 pmol/L (125.8 pg/mL)
Testosterone Tanner stage 3 (2.3-27.0 nmol/L) (66.3-778.7 ng/dL) Tanner stage 4 (6.2-26.4 nmol/L) (178.8-761.4 ng/dL)		6.9 nmol/L (13.3 y) (199 ng/dL)	
Total cholesterol (0-5 mmol/L) (0-193.3 mg/dL)	6.3 mmol/L (243.5 mg/dL)	4.2 mmol/L (162.3 mg/dL)	6.1 mmol/L (235.9 mg/dL)
LDL cholesterol (0-3 mmol/L) (0-115.9 mg/dL)	3.6 mmol/L (139.1 mg/dL)	2.3 mmol/L (88.9 mg/dL)	
HDL cholesterol (1.2-1.7 mmol/L) (46.4-65.7 mg/dL)	0.9 mmol/L (34.8 mg/dL)	0.8 mmol/L (30.9 mg/dL)	
Triglyceride (0-1.7 mmol/L) (0-150.4 mg/dL)	4.01 mmol/L (354.9 mg/dL)	6.26 mmol/L (554 mg/dL)	
HbA1c (20-41 mmol/mol) (4-5.9 %)	32 mmol/mol (5.1%)	35 mmol/mol (5.4%)	43 mmol/mol (6.1%)

Abbreviations: HDL, high-density lipoprotein; LDL, low-density lipoprotein.

Next-generation sequencing of the coding region of genes in the Cambridge Obesity Gene Panel (https://nhsgms-panelapp.genomicsengland.co.uk/panels/130/v4.0) detected a novel heterozygous c.791A > C, p.(N264T) variant in *GNAS*. The variant was confirmed with Sanger sequencing, classified as likely pathogenic (American College of Medical Genetics and Genomics and Association of Clinical Genomic Science guidelines), and was also identified in her mother and brother. This variant has not been reported in known disease (ClinVar and Human Gene Mutation Database) and population databases (1000 Genomes, ESP, ExAC, and GnomAD).

### Case 2 (Proband's Brother)

**Figure 3.**
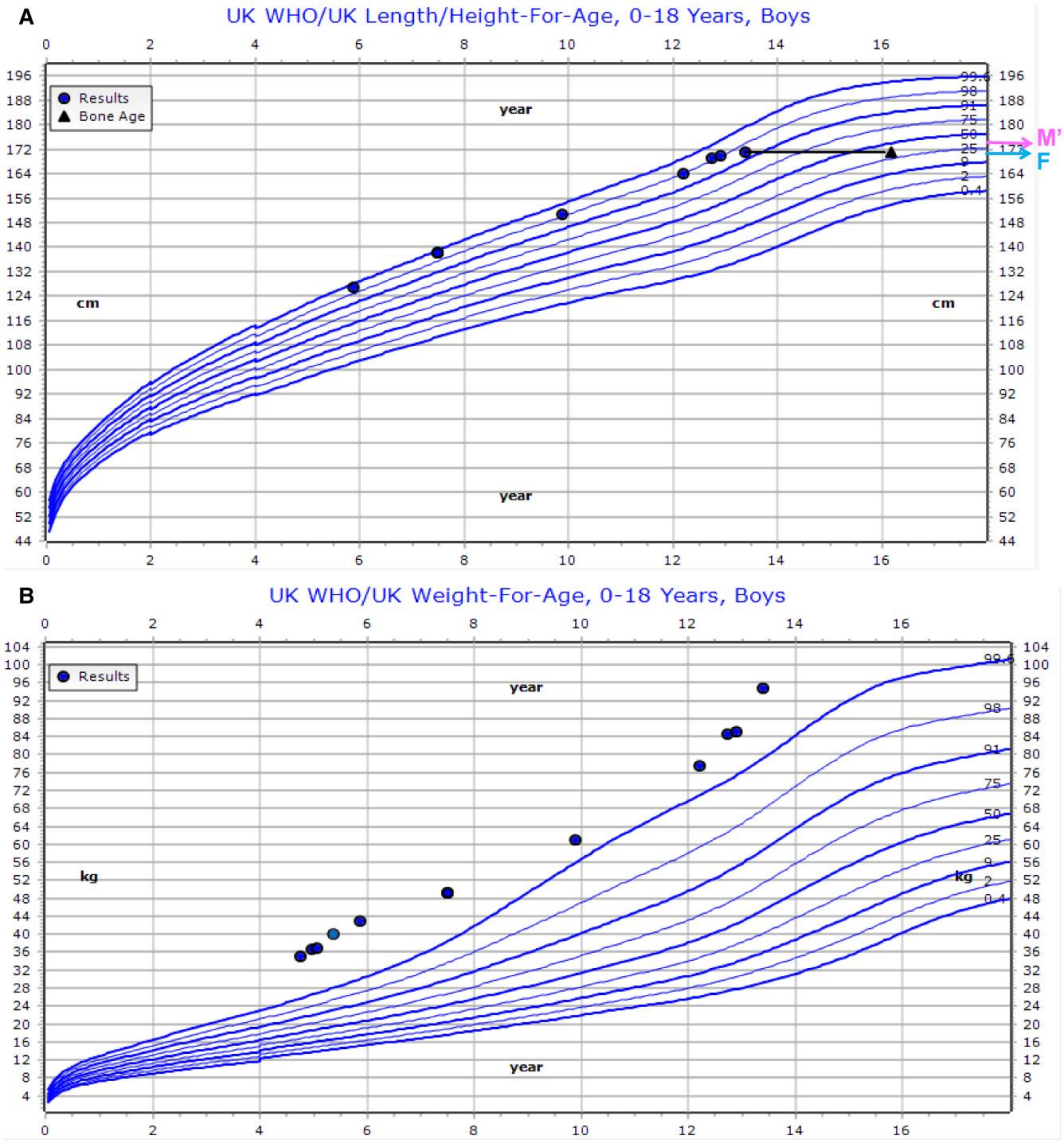
Growth charts depicting height (A) and weight (B) of case 2. Triangles in the height chart depict bone age. X axis represents age in years. Y axis represents height (cm) or weight (kg). M′ indicates height of mother, corrected for sex; F indicates height of father.

The 12-year-old brother was followed in a General Pediatric Obesity clinic from age 3 years. His birth weight was 3 kg (−1.3 SDS). He started walking and talking around 24 months of age and had undescended testes requiring orchidopexy. He had behavioral problems and autistic spectrum disorder. He developed hyperphagia and excessive weight gain from 3 years of age and was referred to a general pediatric obesity clinic. No in-depth endocrine investigations were performed. At the first assessment in an endocrine clinic (aged 12.7 years), he weighed 84.5 kg (+3.1 SDS), measured 168.1 cm (+2.1 SDS) (BMI 29.55 kg/m^2^, +2.9 SDS), HC 56.8 cm (+0.7 SDS). He had mild brachydactyly but no cafe au lait macule. He did not allow pubertal examination (Fig. 3).

#### Investigations

**Figure 4.**
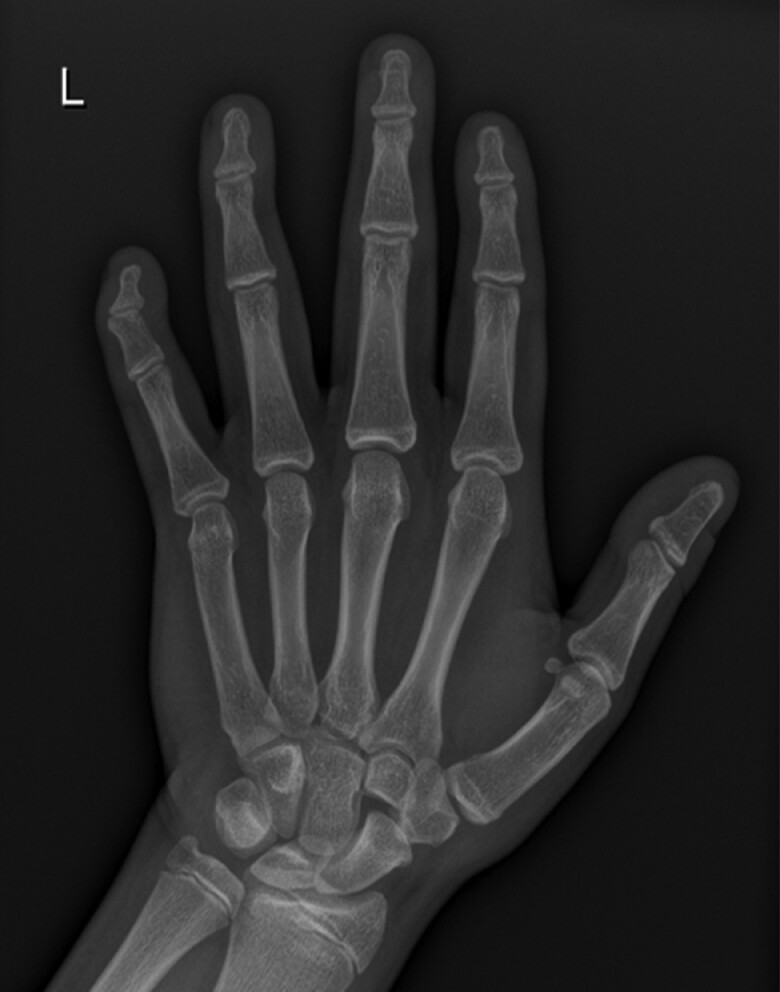
X-ray of the hand and wrist of case 2 showing slightly short metacarpals compared to proximal phalanges; flexion deformity in the distal interphalangeal joint of little finger.

PTH was 18.4 pmol/L (1.6-6.9) (173.5 pg/mL [15.1-65.1]), with normal calcium, phosphorus, and vitamin D but there was no evidence of other hormone resistance. He had hypertriglyceridemia and mild diffuse fatty liver and multiple gallstones. Bone age at 13.4 years was 16.2 years (Fig. 4).

### Case 3 (Proband's Mother)

The 37-year-old mother reported excessive weight gain since pregnancy. She weighed 100 kg (BMI 39.0 kg/m^2^), had normal height (160.2 cm, −0.6 SDS) and HC (55.5 cm (+0.1 SDS) (3). She had mild brachydactyly, which she had not noted herself. She denied a learning disability, was an average student at primary and secondary school, and obtained a diploma for secondary school, after which she left education. She had no hyperphagia and she had been able to lose 15 kg previously, which she regained. She reported that her mother and her siblings were lean and had no hormone conditions nor a learning disability. Mother carries the same *GNAS* variant as 2 of her 3 children. She had mild hypocalcemia with PTH resistance and normal TSH, IGF-1, and gonadotropins, and slightly raised HbA1c and hypercholesterolemia (Table 1).

## Diagnostic Assessment

### Transfection and cAMP Assay

HEK293 cells ATCC CRL-1573TM were transfected using polyethyleneimine (Polysciences) with the pGLO−22f biosensor (Promega) 3xHA-GHRHR (cDNA), 3xHA-MC4R or 3xHA-PTHR (Genscript) as well as WT-Gs (Genscript) or Mutant-Gs (Genscript). A total of 35 000 cells were plated (white-walled, white-bottomed 96-well plates [Greiner] coated with poly-D-lysine [Merck]). Forty-eight hours after transfection, media was removed, cells were washed with assay buffer: 1× HBSS (pH7.4), 24 mM HEPES, 0.1% (w:v) BSA, 3.96 mM NaHCO3, 1 mM MgSO4, 1.3 mM CaCl2, and replaced with 90 µL of assay buffer with 0.45 mg/mL firefly D-Luciferin (Nanolight Technology) and equilibrated for 1 hour (28 °C). Bioluminescence was measured using the BMG CLARIOstar Plus plate reader (BMG Labtech). Prior to the addition of ligand with PTH (Genscript), GHRH (Prospec Bio), or ɑ-MSH (Tocris), 6 basal reads were recorded; an average of these was used to calculate percentage increase over the basal. Readings were taken for 30 minutes with a 0.42-second integration time with no lens.

### Functional characterization of p.(N264T) *GNAS*

**Figure 5.**
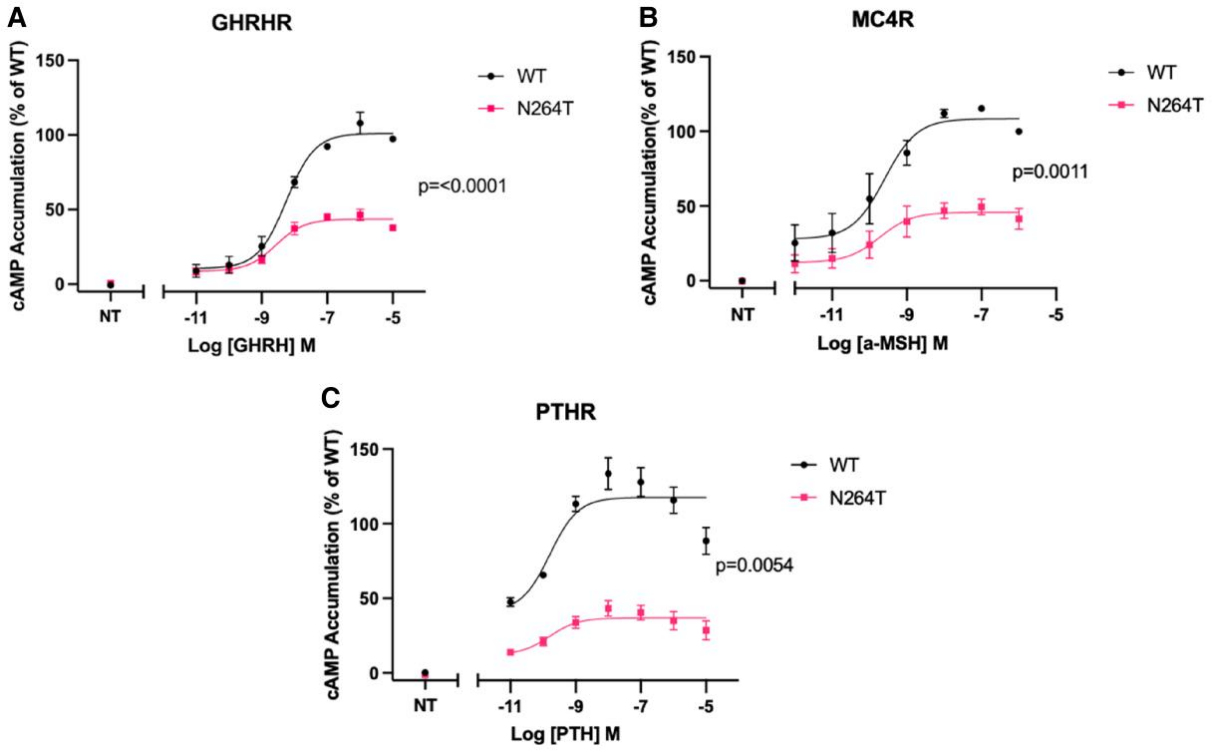
The functional impact of the N264T mutation on cAMP signalling in transiently transfected HEK293 cells. (A) cAMP generation through the GHRHR is significantly impaired in the presence of the N264T mutation. (B) cAMP generation through the MC4R is significantly impaired in the presence of the N264T mutation. NT represents the nontreated control. (C) cAMP generation through the PTHR is significantly impaired in the presence of the N264T mutation. Data are expressed as a percentage increase over the average basal values. Area under the curve of these normalized values were taken and plotted as a concentration response curve. These values were normalized to a percentage of the WT-Gs transfected condition for each receptor where 100% represents the value at the highest concentration of ligand and 0% is the vehicle control condition. Data were fit to a 3-parameter logistic fit in GraphPad Prism. Each plotted data point represents the mean ± SEM of 3 biologically independent experiments performed in technical triplicate. *P* values are derived from an unpaired *t*-test of comparing EMax values from each biological replicate. NT represents the nontreated control.

We evaluated functionality of the *GNAS* mutant by assessing signalling of the Gαs-coupled receptors MC4R, PTHR, and GHRHR as described previously. Maximal ligand-induced cAMP production (Emax) of the GHRHR, MC4R, and PTHR were significantly reduced in the presence of the N264T Gsɑ compared to equivalent cells transfected with the wild-type Gsɑ (Fig. 5).

## Treatment

Liraglutide is licensed for treatment of young people weighing more than 60 kg and aged 12 years with a BMI equal or greater than the equivalent to 30 kg/m^2^ in adults. Case 2 is therefore eligible for glucagon-like peptide receptor agonist treatment, but this is currently unavailable.

## Outcome and Follow up

Case 1, at age 10.2 years, weighs 71.7 kg (+3.2 SDS) and measures 144.5 cm (+0.5 SDS) (BMI 34.3 kg/m^2^ [+3.6 SDS]). Case 2, at age 13.3 years, weighs 94.7 kg (+3.3 SDS) and measures 171.1 cm (+1.4 SDS) (BMI 32.3 kg/m^2^ [+3.1 SDS]).

## Discussion

We describe a novel heterozygous inactivating *GNAS* variant c.791A > C, p.(N264T) resulting in hyperphagia, obesity, developmental delay, mild brachydactyly, a variable effect on height and head circumference, and biochemical mild PTH resistance, fulfilling criteria for a diagnosis of AHO and PHP1A/IPPSD2 in 2 siblings and a similar phenotype without cognitive impairment and normal height in their mother, the phenotype cosegregating in the pedigree.

**Figure 6.**
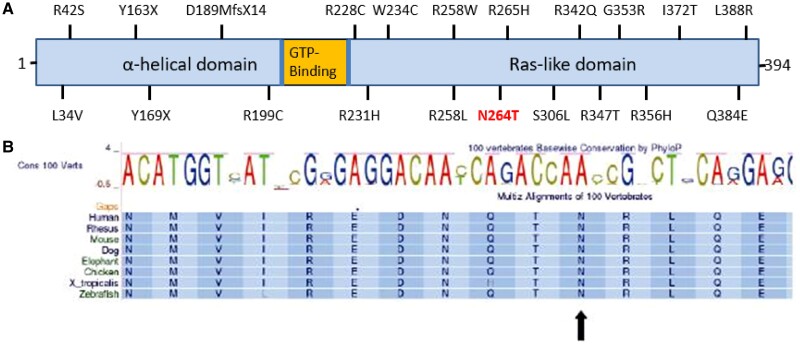
(A) Cartoon (not to scale) depicting the location of N264T in relation to other *GNAS* mutations detected in obese patients in the GOOS study (2). (B) Amino acid sequence of GNAS protein around amino acid 264 in several species showing high degree of conservation across species of the region and amino acid N at 264.

*GNAS* variants were also recently found in patients in GOOS in whom a diagnosis of PHP1A/iPPSD2 had not been previously considered, the majority in the RAS-like domain, as is p.(N264T) (1) (Fig. 6A). Residues 236-296 of the GNAS protein are responsible for the activation of adenylyl cyclase, and thus cAMP production after GPCR activation (4). The variant c.791A > C, p.(N264T) affects a highly conserved amino acid residue 264 (Fig. 6B) and has not yet been reported in any disease or population database. Another missense mutation p.N264H, in which the asparagine is replaced by histidine, is classified as pathogenic and reported in a child with AHO (5). Functional studies of the *GNAS* variants detected in the GOOS study showed impaired coupling to the GPCR and/or defective cAMP production (1). Our in vitro data confirm the functional effect of the N264T variant with reduced cAMP production mediated by PTHR, GHRHR, and MC4R.

Of the 22 GNAS mutations detected in GOOS participants, 5 had brachydactyly and 2 had evidence of TSH or PTH resistance with onset at 8 and 26 years. In our family, both siblings and the mother had mild brachydactyly and all had mild PTH resistance but only the mother had hypocalcemia. There was no biochemical TSH or GH resistance. This report highlights the importance of PTH assessment in obesity, and regular monitoring to detect development of hormone resistance in PHP1A/iPPSD2. Absence of hormone resistance in patients with obesity does not exclude *GNAS* variants.

Obesity in PHP1A/iPPSD2 was previously thought to be due to decreased resting energy expenditure in childhood and not always related to hyperphagia (5). MC4R is dependent on Gsα for downstream activation and production of brain derived neurotrophic factor and transcription factor single minded-1, both involved in appetite and satiety. The MC4R pathway also regulates insulin sensitivity and metabolism (6). Many GOOS participants with *GNAS* variants showed impaired MC4R signalling, suggesting centrally MC4R-mediated hyperphagia leading to obesity (1). Our index case had hyperphagia and obesity in line with impaired MC4R signalling that we found in vitro. Several other mechanisms might contribute to obesity in PHP1A/iPPSD2, such as impaired signalling through B2 and B3 adrenoceptors and thyrotropin resistance–related reduced metabolism (1). There is evidence of improvement in resting energy expenditure with age leading to less pronounced obesity in adults, in line with the successful weight loss in our proband's mother (7). Of note, the proband was able to maintain weight to some extent with individualized support but when that was stopped, she gained weight rapidly. This shows that individualized close support can improve weight, similar to what is seen for some patients with nutritional obesity.

In conditional *GNAS* knockout mice, treatment with MC4R agonists does not increase resting energy expenditure because of the downstream impact of GNAS deletion after MC4R stimulation (6). However, in addition to Gsα, MC4R stimulation can lead to downstream activation via Gqα and G11α (8). Mice studies have demonstrated this pathway in the paraventricular nucleus of hypothalamus mediating food intake. Various ligands binding MC4R can preferentially elicit either Gsα-mediated response or Gqα/G11α-mediated response (8, 9). Setmelanotide, a highly selective MC4R agonist with a preferential Gqα/G11α pathway stimulation, is approved for specific causes of monogenic obesity upstream of the MC4R (pro-opiomelanocortin deficiency, proprotein convertase subtilisin/kexin type 1, leptin receptor deficiency) (9). Its selectivity reduces the side effect profile and the preferential Gqα/G11α stimulation makes it likely effective in children with GNAS defects.

*GNAS* mutations can variably impact height as impaired MC4R signalling can lead to obesity with tall stature, whereas impaired GHRH signalling results in GH deficiency and short stature. The bone phenotype in PHP1A/iPPSD2 can also lead to short stature. Most patients with *GNAS* variants in the GOOS study were growing along the higher centiles before the age of 12 years, similar to other obese patients (2). Our siblings’ growth pattern was similar, whereas the mother was not tall. This report highlights again that *GNAS* variants should also be considered in patients that have normal or tall stature, not displaying the classical phenotype with short stature of PHP1A/iPPSD2. Patients with *GNAS* variants in the GOOS study with impaired GHRH signalling had an impaired pubertal growth spurt. The growth chart and advanced bone age of our proband's brother suggests he may have an impaired growth spurt, in line with our in vitro data of GHRH resistance. The undescended testes could have been a sign of hypogonadotropic hypogonadism but there was no biochemical hypogonadism present.

## Learning Points

- PHP1A/iPPSD2 is a disorder with large clinical heterogeneity.
- Mild PHP1A/iPPSD2 may be missed in general paediatric/obesity clinics.
- Clinical, biochemical, and genetic investigations for PHP1A/ iPPSD2 should be considered in patients presenting with obesity, even without classical signs of PHP1A/iPPSD2.
- These steps may reduce diagnostic delay in PHP1A/iPPSD2.

## Data Availability

Original data generated and analyzed for this case report are included in this published article.
